# Rapid cell separation with minimal manipulation for autologous cell therapies

**DOI:** 10.1038/srep41872

**Published:** 2017-02-02

**Authors:** Alban J. Smith, Richard D. O’Rorke, Akshay Kale, Roberts Rimsa, Matthew J. Tomlinson, Jennifer Kirkham, A. Giles Davies, Christoph Wälti, Christopher D. Wood

**Affiliations:** 1 School of Electronic and Electrical Engineering, University of Leeds, Woodhouse Lane, Leeds, West Yorkshire, LS2 9JT, UK; 2 University of Leeds, Department of Oral Biology, Leeds School of Dentistry, Leeds, UK

## Abstract

The ability to isolate specific, viable cell populations from mixed ensembles with minimal manipulation and within intra-operative time would provide significant advantages for autologous, cell-based therapies in regenerative medicine. Current cell-enrichment technologies are either slow, lack specificity and/or require labelling. Thus a rapid, label-free separation technology that does not affect cell functionality, viability or phenotype is highly desirable. Here, we demonstrate separation of viable from non-viable human stromal cells using remote dielectrophoresis, in which an electric field is coupled into a microfluidic channel using shear-horizontal surface acoustic waves, producing an array of virtual electrodes within the channel. This allows high-throughput dielectrophoretic cell separation in high conductivity, physiological-like fluids, overcoming the limitations of conventional dielectrophoresis. We demonstrate viable/non-viable separation efficacy of >98% in pre-purified mesenchymal stromal cells, extracted from human dental pulp, with no adverse effects on cell viability, or on their subsequent osteogenic capabilities.

Separation and enrichment of cells derived from bone marrow, fat or even surgical waste (e.g. aspirate) are essential for a growing number of modern surgical cell-based therapies including in the treatment of diabetes^1^, in vascular surgery^2^ and in treatments for musculoskeletal disease^3^. A significant limitation to the success of these therapies is in ensuring the viability of transplanted cells, which varies significantly according to harvest method^4^,^5^,^6^,^7^,^8^. The release of cytokines from dead/dying cells induces an inflammatory response that can increase rejection rates and affect the differentiation of transplanted stem cells^9^, making a viable-cell enrichment step critical. Commercial cell separation is based predominantly on three approaches: adherence, density or antibody-binding^10^. Adherence is rarely used clinically owing to extremely low selectivity. Centrifugation also has limited selectivity but is very high-throughput and used routinely where specificity is not critical^11^. Highly-specific separation is achieved using e.g. magnetic activated cell sorting (MACS)^12^, or fluorescence-activated cell sorting (FACS)^13^, in which antibodies against cell-specific markers are conjugated with iron-oxide-containing microbeads or fluorescent labels, respectively. However, these suffer from significantly increased costs and can cause localised tissue damage through e.g. endocytosis of the magnetic particles^14^. Both techniques require the identification of *unique* markers for which antibodies are available, and even where this is possible, binding to a cell surface marker (often a signalling molecule) can trigger an intracellular signalling cascade and alter the cell phenotype. To address these challenges, label-free, microfluidics-based ‘lab-on-a-chip’ technologies including micro-scale filters/pillars^15^, field-flow-fractionation^16^, acoustophoresis^17^, and dielectrophoresis^18^, *inter alia*, are increasingly of interest.

Dielectrophoresis (DEP) employs non-uniform electric fields to exert forces onto suspended cells, whose magnitude and direction are described by the Clausius-Mossotti relation and depend on the polarizability of the cell with respect to the surrounding medium^19^,^20^,^21^,^22^. Cells which are more polarizable than the medium will move to regions of high electric-field gradient (positive, pDEP) and *vice versa* (negative, nDEP), hence differences in polarizability between e.g. viable and non-viable cells^23^,^24^,^25^, cancerous and healthy cells^26^, and blood cells^27^, can be exploited for their separation. However, electrodes necessary for DEP – whether physical, optically patterned^28^ or formed from high-conductivity buffer regions^29^ – must be in contact with, or capacitively coupled to, the medium. To avoid adverse electrochemical reactions and electrode fouling/corrosion, either the fluid conductivity must be low (c.f. typically 0.001–0.020 Sm^−1^), the applied DEP bias must be low (at the expense of significantly reduced throughput)^30^, or the separation regime is restricted to nDEP-based field flow fractionation^16^ comprised of either large (10′s cm), or complex, 3D fluidic channel networks^31^. The otherwise considerable clinical potential of DEP is severely restricted by these limitations, which adversely affect cell viability through Joule heating, pH gradients, or by imposing large osmotic pressures across cell membranes.

Conversely, acoustophoresis can be used to impose high-throughput, *density*-based separation of suspended particles using acoustic radiation forces, produced by pressure waves within the fluid. The pressure waves are generated using a type of surface acoustic wave (SAW) commonly referred to as a Rayleigh-SAW (RSAW), in which a surface-propagating mechanical wave is established in a piezoelectric material (typically LiNbO_3_) by application of high-frequency signals to interdigitated transducers formed on the substrate surface. The RSAW contains a significant surface-normal mechanical component that couples strongly into longitudinal pressure-waves within any overlaid fluid, which, under appropriate conditions, can induce pressure-driven, density-based separation on particles suspended within the liquid. However, the selectivity of density-based separation is limited (i.e. two cells of similar size and density cannot be discriminated).

Remote DEP offers a solution to all of these challenges. Unlike RSAWs, in remote DEP, *lateral* mechanical oscillations are established which propagate along the substrate surface in the form of shear-horizontal surface acoustic waves (SH-SAWs). Two counter-propagating SH-SAWs combine to form a standing wave, creating a localised, non-uniform alternating electric field arising from the compression and rarefaction of charge-centre separations within the crystal lattice. This electric field induces DEP in cells suspended within an overlaid microfluidic channel (Fig. 1). By locating the SAW electrodes externally to the fluidic channel, no physical electrodes are present in the fluid. Instead, *virtual* electrodes are formed, with a pitch and periodicity equivalent to those of the externally located IDTs, but which cannot constitute an electron source, and therefore electrochemical reactions (e.g. oxidation, reduction and radical formation), which lead to electrode fouling, bubble formation, heating, cell damage, pH gradients, *inter alia*, are prevented. Furthermore, this means that cell separation is not limited to low-conductivity medium and can be achieved in conductivities >1 Sm^−1^ if required. Unlike traditional RSAW-based particle manipulation^32^,^33^,^34^, using a horizontally-polarized SH-SAW minimises acoustic pressure wave generation within the fluid by elimination of the surface-normal mechanical wave component. Therefore the cells are only acted upon by DEP forces, causing them to align at regions of high and low field gradient, depending primarily on the relative polarizability of the cells and medium; SAW-DEP selectivity is hence optimised through careful choice of medium conductivity and SAW operating frequency, allowing their relative polarizabilities to be tuned. This technique therefore combines the high-throughput capabilities of acoustophoresis, with the exquisite discriminatory capabilities of DEP, and offers a possible solution to a wide range of current cell-separation challenges.

## Results

### Applicability of SAW-DEP for eukaryotic cell separation

**Figure 1:**
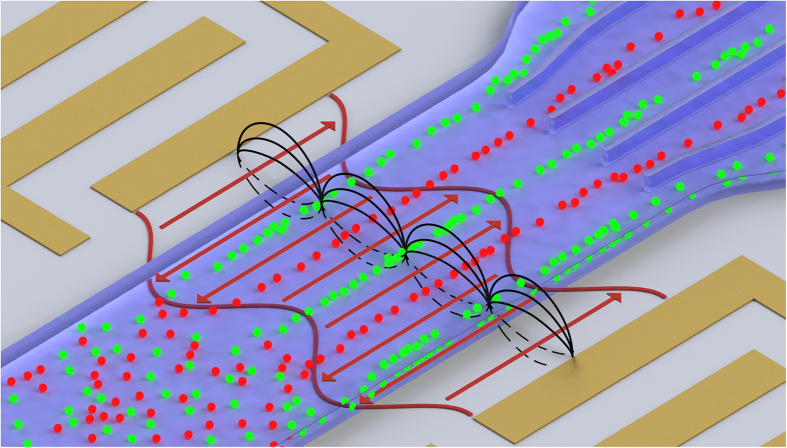
SAW-DEP separation mechanism. Schematic of a SAW-DEP device separating two cell types for subsequent collection. The SAW-induced ac electric fields, generated by opposing interdigitated transducers, are depicted as black lines, and the lateral mechanical oscillations are in red, indicated by arrows. Careful choice of the SAW frequency and fluid conductivity causes cell type 1 (red) to experience negative DEP, and therefore align to areas of low field gradient. Cell type 2 (green) conversely experience positive DEP and align to regions of highest field gradient.

We have employed SAW-DEP for rapid, non-destructive separation of viable from non-viable cells, to demonstrate the potential of the approach for next-generation cell therapies using minimally manipulated cells. To study the operating principle, we first investigated the separation of viable from non-viable *Saccharomyces cerevisiae* yeast cells. The SAW-DEP device comprised a two-port, linear microfluidic polydimethyl siloxane (PDMS) channel 1000 μm wide and 50 μm deep, formed on a lithium tantalate substrate (Materials and Methods). Interdigitated transducers (IDTs), used to establish the SH-SAW standing wave, are located on opposite sides of the fluidic channel (Fig. 1) creating an active SAW-DEP region 1 mm in width along the channel. First, the device insertion loss was assessed as a function of buffer conductivity using a 2-port network analyser, and was found to be constant at −20.5 ± 0.5 dB (corresponding to a SAW-power of 5 mW per IDT under application of 500 mW power to each transducer) across six orders of magnitude in conductivity (0.0001–10 Sm^−1^), indicating that even in high conductivities, no acoustic energy is dissipated into the fluidic system through, for example, Joule heating (Supplementary Fig. S1). To support this, we performed full 3D finite element simulations of the device (see Supplementary Fig. S2; Materials and Methods) for fluid conductivities in the range 0–1 S/m, for a SAW power of 10 mW (double the maximum power used during our experiments). The results (Supplementary Fig. S3) show a small increase in temperature (<0.5 °C above ambient) for medium conductivities below 0.2 S/m (as used in this paper), and only 3.5 °C for a conductivity of 1 S/m – well within safe limits for maintaining cell viability.

**Figure 2:**
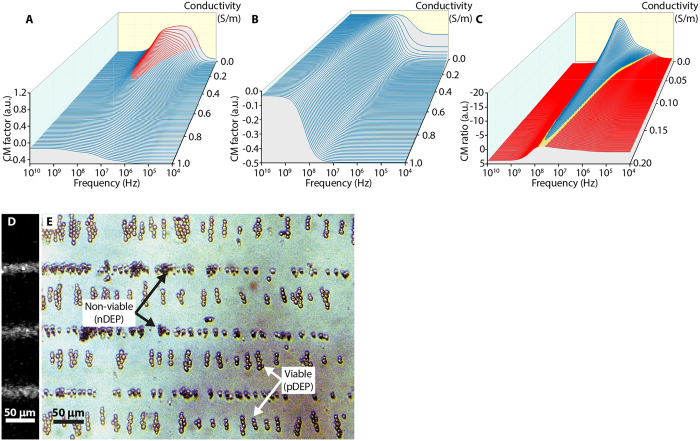
Clausius-Mossotti calculations and yeast-cell separation. Calculated CM factors for (**A**) viable and (**B**) non-viable yeast cells as a function of SAW-DEP frequency and media conductivity. Red lines correspond to positive values of CM factor (pDEP) and blue to negative (nDEP). (**C**) Ratio of viable:non-viable CM factors, where a ratio of between −0.95 and −1.05 (green) corresponds to conditions under which the pDEP and nDEP forces are approximately equal and opposite. (**D**) Image of latex beads, which experience nDEP under all tested conditions, used to calibrate the location of nDEP alignment within the channel. (**E**) An optical micrograph of live and dead yeast cells separated using a SAW-DEP device, operating at 20 MHz in 0.15 S/m buffer conductivity. Dead cells, stained with Trypan blue, align at regions of low electric field gradient (nDEP) owing to their reduced polarizability with respect to the surrounding medium, and *vice versa*. Pearl chaining of the live cells along the electric field lines occurs owing to mutual DEP and dipole-dipole interactions.

A live culture of *S. cerevisiae* was separated into two populations, one of which was subjected to prolonged exposure to isopropanol to ensure complete cell death. Dead cells were stained using Trypan blue before recombining in approximately equal proportion with the live culture. The Clausius-Mossotti (CM) factors were calculated as a function of medium conductivity for viable (Fig. 2a) and non-viable (Fig. 2b) cells, using a two-shell model^35^ (Materials and Methods). Solutions to the model lie between upper (pDEP) and lower (nDEP) bounds of 1 and −0.5, respectively, where the cross-over between pDEP and nDEP occurs at a CM factor of zero. To optimise cell separation, the ratio between viable:non-viable CM factors (Fig. 2c) should therefore be close to −1, corresponding to conditions in which the pDEP and nDEP forces are equal and opposite. The region corresponding to CM ratios between −0.95 and −1.05, shown in green in Fig. 2c, provides a range of suitable device operating frequencies and media conductivities, from which values of 20 MHz and 0.15 Sm^−1^ were chosen, respectively. Although operation at higher frequencies would allow the use of higher medium conductivities, the correspondingly reduced spatial separation of the pDEP and nDEP regions (which is proportional to the SAW wavelength) would complicate subsequent extraction of the separated cells.

The position at which nDEP occurs within the fluidic channel was first calibrated using polystyrene beads, which experience nDEP for all conductivities above 1 MHz SAW-DEP operating frequency (Materials and Methods; Supplementary Fig. S4). The beads were imaged using a fluorescence microscope (Fig. 2d), before the mixture of viable and non-viable yeast cells were introduced (Fig. 2e). The stained dead cells aligned at regions of low electric field gradient (nDEP) owing to their low polarizability in comparison with the surrounding fluid. Conversely, the viable cells were seen to align at regions of high electric field (pDEP). A small proportion of cells were not aligned owing to adhesion to the substrate/fluidic channel walls. The ‘pearl chaining’ of live cells in the pDEP regions arises from a combination of mutual dielectrophoresis, in which a localised attractive DEP force is imposed on neighbouring cells arising from distortion of the surrounding SAW-induced electric field and dipole–dipole interactions, which cause the mutually attracted cells to align along the SAW-induced electric field lines^36^. These effects occur typically at regions of highest field intensity and serve as further confirmation that live cell alignment is dominated by pDEP. Statistical analysis of approximately 10,000 cells revealed that, of the cells present in the original mixture, 97% were separated successfully into either nDEP or pDEP regions, with the remaining cells adhering to the channel walls/substrate. Within the pDEP regions, significant enrichment (>99%) of viable cells was observed (Supplementary Fig. S5). A maximum separation throughput of 10^4 ^cells/min was observed, which is approaching current FACS-based sorting speeds. However, it should be noted that this was achieved on a single channel, prototype device using a technology that is inherently scalable.

Upon increasing the conductivity of solution, such that all cell-types should have a negative CM factor, all cells were observed to align within the nDEP regions, verifying that cell alignment is dominated by electrical DEP-forces (as opposed to acoustophoretic forces). To quantify this claim, 3D finite element simulations were performed in which the DEP-based forces were isolated from the acoustic radiation forces. The results (Supplementary Fig. S6) show than any acoustic radiation forces induced by the SH-SAW standing wave, do not contribute to cell separation.

### Mesenchymal Stromal Cell separation

Adult mesenchymal stromal cells (MSCs) are a mixed cell population containing stem cells with the potential to differentiate along a range of lineages, including osteoblastic for the ultimate production of bone. Such cells are expected to play an important role in next generation regenerative cell therapies^37^,^38^,^39^. For this work, human MSCs obtained from dental pulp (dental pulp stromal cells, DPSCs), were harvested from extracted impacted third molars obtained from three donors, and were cultured in basal medium (Materials and Methods).

**Figure 3:**
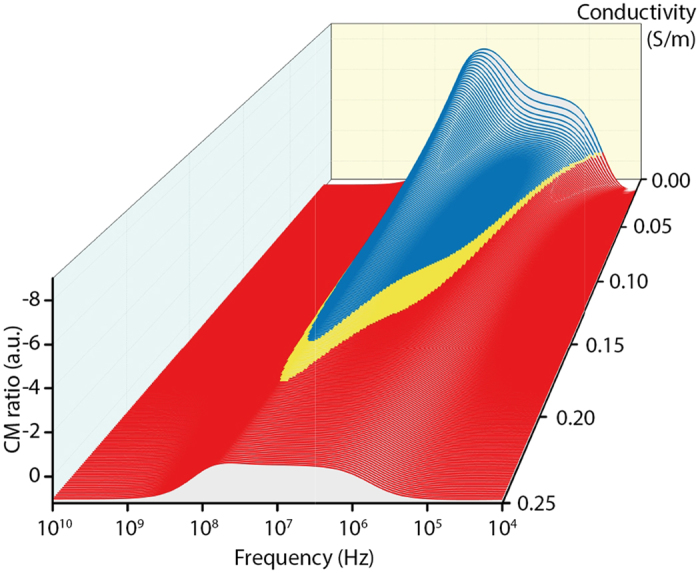
CM ratio for human erythrocytes. Calculation of the CM ratio for human erythrocytes ratio was used as an estimate to allow choice of operating conditions for the subsequent separation of MSCs used in this study.

**Figure 4:**
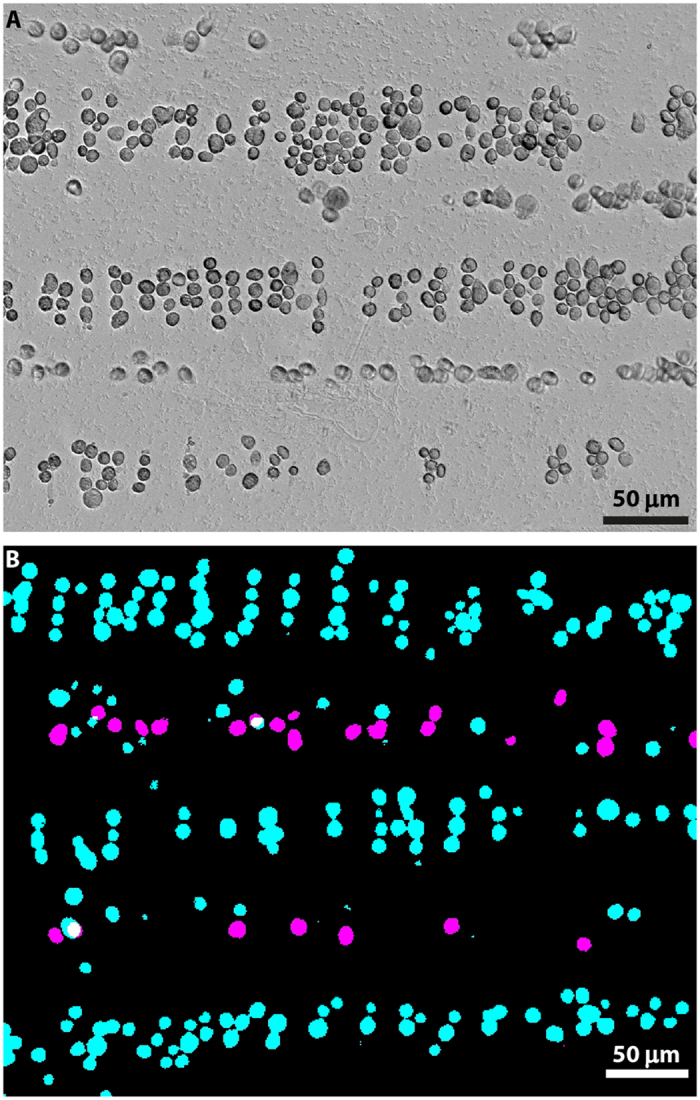
Separation of viable from non-viable DPSCs at 10 MHz. (**A**) an unstained brightfield image in media conductivity of 0.15 S/m. (**B**) A fluorescence image of Calcein-AM stained viable cells (Cyan) overlaid with PI-stained non-viable cells (purple) in a media conductivity of 0.15 S/m.

The dielectric properties of DPSCs are not available in the literature; therefore a single-shell model of the CM factors for viable and non-viable human erythrocytes was employed as a first approximation^40^ (Materials and Methods). These calculations, shown in Fig. 3, suggest that good separation would be achieved in a SAW-DEP buffer conductivity of ~0.15 Sm^−1^ at an operating frequency of 10 MHz. Cultured DPSCs were therefore buffer exchanged and resuspended in SAW-DEP medium (Materials and Methods), pH 7.3, and which had a final measured conductivity of 0.15 Sm^−1^. A 25 μL sample volume, containing ~250,000 individual DPSCs was used in each experiment. These were introduced into the sterilised fluidic channel at a continuous flow rate of 3 μL/min. The cells were therefore present within the fluidic channel for ~8 minutes and 20 seconds but owing to the channel geometry and flow rate, each cell was subjected to the SAW-DEP electric field for a maximum of 1 second. This was sufficient time to allow continuous-flow separation at an applied SAW power of 7 dBm per IDT (~5.0 mW at 50 Ω impedance), accounting for the device insertion loss. The alignment of cells for each donor was determined from optical images (e.g. Fig. 4a) recorded at 30 second intervals throughout each separation procedure, and showed cell alignment in nDEP regions of 17%, 34.1% and 14.7% of the total sample populations for donors 1, 2 and 3, respectively. The high nDEP alignment of cells for donor 2 was attributed to the advanced age of the cells (passage 9) in comparison with all other measurements (passage 3–5), which could manifest itself as a decrease in population viability. However, nDEP-alignment alone is not necessarily directly indicative of a cell’s viable state and the DPSCs from later passages may have had an altered phenotype.

In order to assess the viability of cells within the pDEP/nDEP regions directly, the process was repeated using fluorescently-labelled DPSCs (Materials and Methods). Dead cells were stained using propidium iodide (PI) (Fig. 4b, purple) and live cells using a green Calcein-AM stain (Fig. 4b, cyan). The cells in the pDEP region were seen to be entirely viable, corresponding to >98% enrichment (a very small number of dead cells were occasionally observed within the pDEP region, which is attributed to cell–cell adhesion). Furthermore, a number of live cells were also seen to lie within the nDEP regions. This is attributed to a discrepancy between the optimum separation conditions for DPSCs, and the estimation obtained by using CM calculations for human erythrocytes. Binary separation of viable from non-viable DPSCs can be achieved at 10 MHz operating frequency by reducing the medium conductivity to 0.05 S/m (Supplementary Fig. S7), indicating that a lower operating frequency is required for optimum separation in high conductivity medium for this cell type. However, although the high-conductivity separation was not optimised for DPSCs, significantly high (>98%) enrichment of viable cells within the pDEP regions was still achieved; the benefit to the cells of maintaining a higher conductivity medium therefore outweighs the loss of a small proportion of viable DPSCs.

### Characterisation of DPSC separation

Following separation, the viable and non-viable DPSCs were recombined, collected and diluted in PBS before centrifugation and re-suspension in PBS for subsequent viability tests. Propidium iodide (10% dilution) was added and cells were incubated at room temperature for ~10 minutes to stain the dead cells within the mixture. The viable/non-viable cell populations were then analysed using a BD LSRII at 488 nm excitation wavelength. Forward (FSC) and side scatter (SSC) measurements were used to generate a gate for the removal of cellular debris from the analysis.

**Figure 5:**
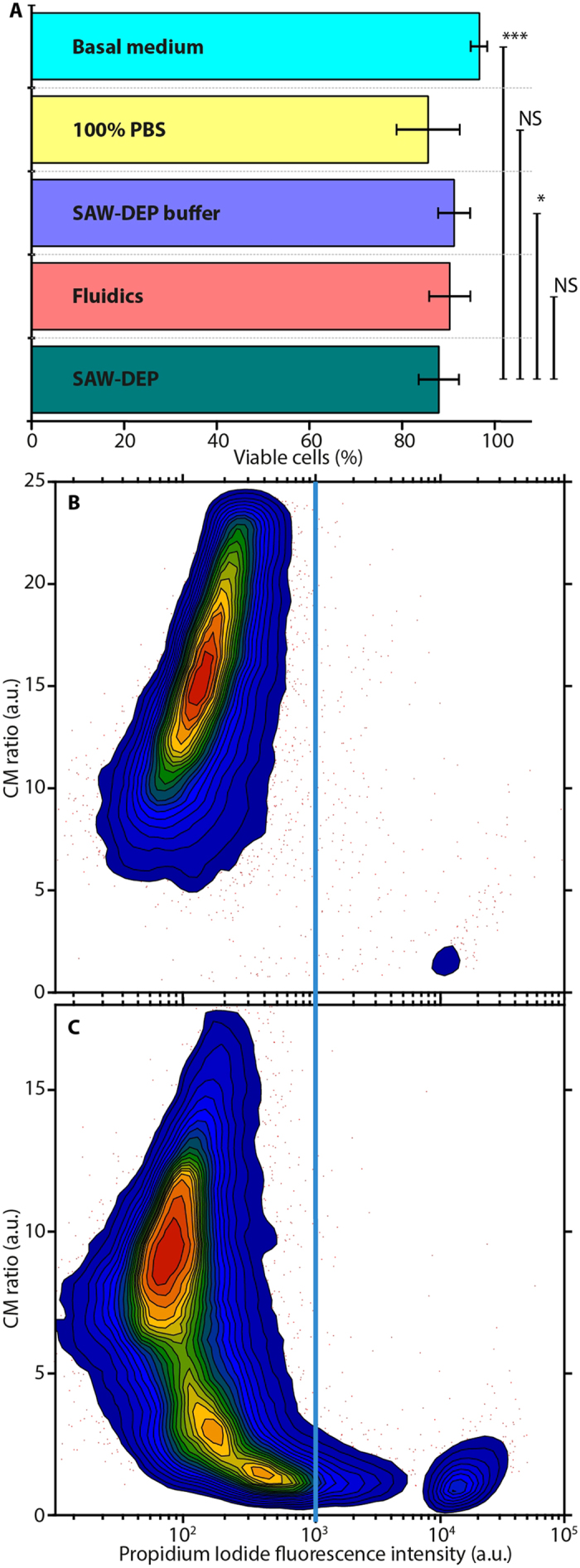
Flow cytometry analysis of post SAW-DEP DPSC viability. (**A**) Mean viability and standard deviation (n = 9) for DPSCs subjected to different conditions, measured using flow cytometry (*p < 0.05; ***p < 0.001 using the Mann-Whitney U test; NS, not significant; error bars are standard error, n = 9). Averaged contour plots for cells from Donors 1–3 showing viability counts and standard errors for (**B**) a control group taken directly from the basal medium and (**C**) cells having undergone SAW-DEP separation. Contours are drawn at 10% probability intervals. The vertical line corresponds to the cut-off between viable (left) and non-viable (right) cells.

Cells from the three donors were each divided into five samples. These included three control samples of DPSCs held at room temperature in either basal medium, PBS or SAW-DEP buffer with no exposure to the fluidics system or any electric fields. A further control sample contained DPSCs that had passed through the fluidic system in SAW-DEP buffer but without the application of the SAW-DEP electric field. The final sample comprised DPSCs that had been separated using SAW-DEP and thus exposed to both the microfluidics system and to the electric field. All controls were held in their respective state for a time period equal to that of a typical SAW-DEP separation process (30 minutes, including buffer exchanges) and underwent the same number of buffer exchanges to standardise other variables that may affect the final viability count. Figure 5a shows viability counts obtained using flow cytometry (FC) for each sample. Statistical analysis of each measurement (n = 9) showed that DPSCs separated using SAW-DEP showed slightly decreased viability in comparison with control cells held in basal medium (p < 0.001) and with those held in SAW-DEP separation medium (p < 0.05), though differences in absolute values for viability were small. There was, however, no significant decrease in viability between cells separated using SAW-DEP and those which had been passed through the fluidics system (without an active SAW-DEP field) or those held for the same time period in PBS (no exposure to fluidics or SAW-DEP field). A significant proportion of the observed cell death can therefore be attributed to the duration for which the cells are removed from the basal medium, and which are then exposed to shear stresses when passed through the microfluidic system, rather than to the SAW-DEP separation procedure *per se*.

Contour plots of viability counts, averaged across the donor pool, for cells taken directly from basal medium and cells separated using SAW-DEP, are presented in Fig. 5b and c, respectively. A gate (vertical line, Fig. 5b and c) was placed at a PI intensity of 10^3^, and was maintained for all measurements, separating the viable (left) and non-viable (right) cell populations. Although the majority of DPSCs remained viable following SAW-DEP (87.9 ± 1.5%), there was an 8.8% increase in the proportion of dead cells after SAW-DEP compared with the control sample (96.7 ± 0.4%). Variations between donors/experiments are attributed to cell age and experimental conditions.

### Osteogenic potential of DPSCs after SAW-DEP

**Figure 6:**
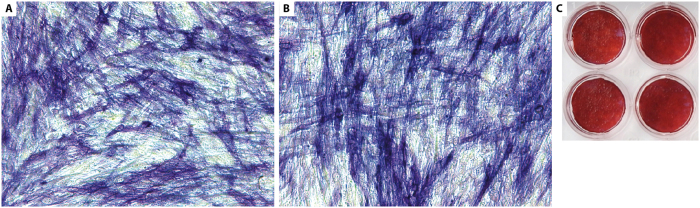
Post SAW-DEP separated DPSC activity. Histochemistry of alkaline phosphatase activity in DPSCs after 2 weeks in osteoinductive culture for (**A**) a control not exposed to SAW-DEP, and (**B**) DPSCs recovered after SAW-DEP. Both demonstrate intense blue staining. (**C**) Alizarin Red staining for mineralisation for the control and SAW-DEP separated cells. No qualitative differences were seen for all tests (n = 6).

DPSCs separated by SAW-DEP were cultured in osteoinductive medium to investigate their ability to differentiate along the osteogenic lineage as demonstrated by the production of mineralised nodules *in vitro*. A control comprising DPSCs in SAW-DEP buffer and passed through the microfluidics but not subjected to SAW-DEP separation, was also used. Following SAW-DEP, DPSCs were seeded into wells at a density of 4 × 10^4 ^cells/cm^2^, and incubated in basal medium at 37 °C in a humidified atmosphere at 5% CO_2_ for 24 hours (Materials and Methods). Osteoinduction was achieved using Complete STEMPRO^®^ Osteogenesis Differentiation Medium, following the manufacturers recommendations. After 8 days, alkaline phosphatase (an early osteogenic marker)^41^ activity was measured for cells in each group (Fig. 6a and b). Finally, after 18 days, Alizarin Red S staining was used with the remaining samples to test for the presence of mineralised deposits in the cultures. The results demonstrated that DPSCs separated using SAW-DEP express alkaline phosphatase and subsequently produce a mineralised matrix, both within the same timescale, and to the same degree as the positive (no SAW-DEP) control (Fig. 6c; Materials and Methods).

## Discussion

In summary, we have demonstrated a new technique for high-throughput, continuous flow separation of viable from non-viable human MSCs, exemplified using DPSCs, which overcomes the limitations of conventional dielectrophoresis. By using shear-horizontal surface acoustic waves to generate virtual electrodes within a microfluidic channel, non-uniform electric fields can be generated within the channel without inducing Joule heating and electrode fouling common to other DEP-based techniques, allowing operation in high-conductivity, physiologically-relevant media. Cells separated using SAW-DEP demonstrate no significant decrease in viability over controls in which the SAW-DEP was inactive and, furthermore, exhibit no detectable reduction in osteogenic differentiation capabilities. A separation throughput of 10^4 ^cells/min has been demonstrated, limited only by the single-channel design of the prototype device, and by the low operating power of the prototype driving electronics. It is anticipated that this can be increased by as much as two orders of magnitude, through scale up and optimisation of the SAW-DEP separation conditions. Although the *in situ* frequency tunability of SAW-DEP is limited by the IDT spacing/wavelength dependence of the generated acoustic wave, the ability to design IDTs of different frequencies, coupled with the ability to select an appropriate medium conductivity from a previously unrealisable conductivity range, allows SAW-DEP to be used to separate a broad range of cell types. This technique therefore has the potential to create a step-change in cell separation for autologous cell-based therapies, operating with minimal cell manipulation and within intra-operative timescales.

## Materials and Methods

### Device fabrication and operation

The SAW-DEP device comprised two sets of opposing interdigitated transducers (IDTs) separated by 1 mm and forming a 1-mm-wide acoustic aperture. IDT finger widths of 100 μm and 50 μm, each with a mark-to-space ratio of 1, were chosen to give SAW operating frequencies of 10 MHz and 20 MHz, respectively, on Y-cut, 42°-rotated LiTaO_3_ (calculated using a datasheet value for the acoustic velocity of 4,022 ms^−1^).

Firstly, a 200 nm Al film was e-beam evaporated onto the LiTaO_3_ surface to act as a surface charge dissipation layer (to counter the pyroelectric nature of LiTaO_3_ during subsequent heating steps) and to act as a sacrificial undercut for subsequent photolithography. Positive photoresist (Shipley S1813) was next deposited and soft-baked by ramping a hotplate to 115 °C at 5 °C/min for a total of 19 minutes before being exposed through an optical mask, and then developed in MF-319. PAN etch, comprising 85% phosphoric acid, DI water, 70% nitric and 100% acetic in a 25:5:1:1 ratio was then used to etch an undercut into the Al and to expose the substrate in the desired pattern. Ti/Au (20/80 nm) was deposited via an e-beam evaporator, and lift-off performed in acetone at room temperature. Finally, remaining Al was removed from the substrate using PAN etch.

Devices were impedance-matched to 50 Ω using lumped-element matching networks, designed in Microwave Office. After matching, insertion losses of −20 dBm dB and −23 dB were recorded for the 10 MHz and 20 MHz devices, respectively.

### Viable/non-viable yeast cell preparation

Dormant *Saccharomyces cerevisiae*, obtained from baker’s yeast granules (Allinsons), was activated in culture medium comprised of 3 g of sabouraud dextrose broth dissolved into 100 mL DI water, heated to 37 °C. Yeasts were pelleted at 10,000 g for ten minutes and buffer exchanged into 25 mL of 0.85% NaCl to halt yeast growth. To ensure high numbers of live cells, experiments were performed within five days of suspension in 0.85% NaCl.

To produce discrete populations of live and dead cells, the suspended yeasts were next concentrated by pelleting at 10,000 g for ten minutes and resuspending in 2 mL of 0.85% NaCl. Of this, 1 mL was diluted into either 24 mL of isopropanol (IPA) (non-viable cells) or 24 mL of 0.85% NaCl (viable cells). Each population was incubated for 1 hour, before pelleting at 10,000 g for ten minutes and resuspending in 2 mL of 0.85% NaCl. The two samples were then washed twice (to remove any residual IPA) by centrifuging at 6,700 g for one minute, then re-suspending in 1 mL of 0.85% NaCl.

A heterogeneous mixture of viable and non-viable cells was next formed by mixing 15 μL of the viable cell solution with 50 μL of the non-viable cell solution and diluting with 435 μL of 100% PBS. An increased volume of non-viable cell solution was used to produce an equivalent number of viable/non-viable cells in the resultant mixture to compensate for dead cell loss during centrifugation.

### Staining of non-viable yeast cells

The mixture of viable and non-viable cells was next mixed in a 1:1 ratio with of 0.04% Trypan blue cell stain, dissolved in 500 μL of saline solution and incubated at room temperature for five minutes. Trypan blue selectively stains the cytoplasm of dead cells owing to increased permeability^42^. Excess stain was removed from the solution by twice centrifuging at 10,000 g and re-suspending in 0.1% PBS, producing a final mixture of viable and non-viable cells in a medium of conductivity 0.16 S/cm (measured using a conductivity meter, Hanna Instruments 8733).

### Ethical approval and informed consent for DPSC use

Purified human MSCs obtained from dental pulp (dental pulp stromal cells, DPSCs), were harvested from extracted impacted third molars from three donors, both male and female, aged between 21–46 years. Extracted teeth were obtained from patients attending Leeds Dental Institute and provided anonymously to the researcher via the University of Leeds School of Dentistry’s “Human Tissue Act (HTA)” -compliant Research Tissue Bank, with approval from Leeds East Research Ethics Committee (NRES REC ref: 07/H1306/95 + 5). All teeth were obtained following written informed consent from the patients and were donated to the Bank for use in research following local approval. Permission to use teeth for the proposed project was secured from the Dentistry Research Ethics Committee, in compliance with the Bank’s policy. Use of samples issued from the Bank is monitored annually by both the Leeds East Research Ethics Committee and the Quality Assurance team at Leeds Teaching Hospitals Trust. The Bank is also subject to external audit by the UK Medicines and Healthcare products Regulatory Authority (MHRA) to ensure all procedures are compliant with the HTA.

### DPSC isolation and culture

DPSCs were isolated using collagenase I (3 mg/mL; Invitrogen, Paisley, UK)/dispase (4 mg/mL; Roche, Mannheim, Germany) digestion^43^ and selected using plastic adherence and colony formation. Cultured cells were maintained in basal medium comprised of α-MEM, 10% foetal calf serum (Sigma Aldrich, Poole, UK), 100 units/mL penicillin, 100 μg/mL streptomycin solution (Sigma-Aldrich, Gillingham, UK) and 1% of 200 mM L-glutamine (Sigma-Aldrich). Cultures were incubated at 37 °C, 5% CO_2_ and routinely passaged before full confluence. DPSCs at passage 2 to 5 were used in all experiments, unless otherwise stated.

### DPSC preparation for SAW-DEP

Following culture as described above, DPSCs were detached from the culture flasks and buffer exchanged to a concentration of 10^7 ^cells/mL, into SAW-DEP medium consisting of sucrose (7.5% wt/vol), dextrose (0.3% wt/vol), bovine serum albumen (BSA, 2% wt/vol), ethylenediaminetetraacetic acid (EDTA, 0.02% wt/vol), HEPES (0.6% wt/vol) and NaCl (0.05% wt/vol). The buffer pH was adjusted to pH 7.3 using KOH, and the final buffer conductivity was measured to be between 0.14–0.16 Sm^−1^ using a Hanna Instruments conductivity meter (HI8733).

### Viable/non-viable DPSC staining for fluorescence imaging

DPSCs were prepared for fluorescence microscopy by separating a cell population into two equal aliquots, one of which was subjected to heat treatment to kill all cells, and one which was not. The two populations were each stained using the procedures outlined below, and then remixed before undergoing SAW-DEP separation, allowing the viability of cells in pDEP and nDEP alignment positions to be assessed.

For imaging, cells were separated using SAW-DEP under zero-flow conditions, allowing two separate images to be captured of the same population, one of the viable (green emission) and one of the non-viable (red emission) DPSCs, which could then be overlaid into a composite image of the viable/non-viable cell alignment. Simple thresholding was applied to each image prior to forming the composite, in order to produce a single colour intensity for each fluorescence, for clarity.

#### PI staining for non-viable cells

For the non-viable cell fraction, mixed viable and non-viable DPSCs were washed twice with DEP buffer before staining using propidium iodide solution (PI; BioLegend, San Diego, CA). PI has a conductivity of ~1.5 S/m in its pure form. In order to adjust the medium conductivity to 0.15 S/m for subsequent SAW-DEP, cells were suspended in a buffer comprised of sucrose (8.5% wt/vol), dextrose (0.3% wt/vol), and BSA (2% wt/vol), producing a medium conductivity of 0.024 S/m. PI was then added (9% vol/vol) which resulted in a final measured medium conductivity of 0.15 S/m. The cells were then left in a 60 °C water bath for 15 minutes, to allow cells to be stained as they were killed.

#### Calcein AM staining for viable cells

Live DPSCs were stained using Calcein-AM (Biotium, Hayward, CA), diluted to a 10 μM concentration in PBS. Adherent cells were first detached using trypsin solution before undergoing a centrifuge–resuspend–centrifuge wash cycle in PBS. The resulting pellet was then suspended in the Calcein–AM solution and left in the dark for 30 minutes to stain the live cells. After staining, cells were washed twice and finally resuspended in DEP buffer, and mixing with 9% (vol/vol) PI, which both adjusted the medium conductivity to the required value of 0.15 S/m, and stained any dead cells which may be present in the live population.

### Flow cytometry protocol

Remote DEP samples were compared to several control samples of DPSCs, all of which were maintained at room temperature in their respective buffers for the duration of a typical SAW-DEP experiment (30 minutes). These included: a control left in culture medium (30 minutes); a control left in PBS; a control in SAW-DEP buffer; and, a control where cells, suspended in SAW-DEP buffer were passed through the same microfluidics as the SAW-DEP sample, but with the electronics/cell sorting capabilities turned off.

To prepare for flow cytometry, 2–5 × 10^4 ^cells in 200 μL were washed by topping up to 1 mL with PBS, centrifuging, discarding the supernatant, and re-suspending in 100 μL of PBS. Propidium iodide (PI, 5 μL) was added to each tube to stain the cells. After 12 minutes, the concentration of cells was adjusted by adding 250 μL of PBS (to a final volume of 350 μL), ready for the flow cytometer.

A BD^™^ LSRII instrument was used for flow cytometry. Samples were excited with a blue 488 nm laser, and fluorescence was collected via a 550 nm long pass dichroic mirror and a 575/26 nm band pass filter.

Forward scatter (FSC) and side scatter (SSC) were measured simultaneously on a contour plot to define a population of intact cellular bodies. A gate was drawn around the first definable population above the background (caused by electrical noise and particulate matter, including cellular debris) such that only intact cellular bodies were examined. This gate was used for all experiments, and 10,000–20,000 events were counted per experiment (generally 60–70% of which were intact cellular bodies). Forward scatter and PI fluorescence (logarithmic scale) were measured simultaneously on a contour plot to define viability. The positive control showed two separate populations, one of which was heavily fluorescent with PI and was assumed to be the dead fraction. The live/dead gate was drawn between these two populations, at a PI fluorescence intensity of 10^3^, to give a realistic viability for the positive control, which was an average of 96.7% (n = 18). This gate was kept the same for all experiments.

### Characterisation of osteoinduced DPSCs

#### Osteoinduction of DPSCs

After DEP, samples were washed once in basal medium and seeded into either 24-well (for alkaline phosphatase staining) or 12-well (for Alizarin Red staining) plates, at a density of 40,000 cells/cm^2^. In both cases, cells were grown in basal medium for 24 h, before switching to a Complete STEMPRO^®^ Osteogenesis Differentiation Medium (A1007201; Thermo Fisher, Paisley, UK) for the required time periods as below.

#### Alkaline phosphatase staining

The STEMPRO^®^ medium was changed and replaced after 4 days, and cells were assayed for alkaline phophatase (ALP) after 8 days by fixing in 70% ethanol for 15 minutes, and staining using a leukocyte alkaline phosphatase staining kit based on naphthol AS-MX phosphate and fast blue RR salt (85L1; Sigma-Aldrich) according to the manufacturer’s instructions.

#### Alizarin Red staining

The STEMPRO^®^ medium was changed and replaced every 4 days for a period of ~18 days, at which point the cells were fixed in 70% (v/v) ethanol for 1 hour, washed with ultrapure water and stained with Alizarin Red solution (Millipore, TMS-008-C) for 10 min. Samples were then washed 5 times with ultrapure water before imaging.

### Dielectrophoretic force and Clausius-Mossotti factor calculations

For the calculation of the Clausius-Mossotti (CM) factor, the cells are assumed to be homogeneous dielectric spheres, broken into ‘shells’ to reflect their internal structure (see below). The dielectrophoretic force acting on such a sphere is given by:


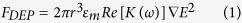


in which *ε*_*m*_ is the permittivity of the surrounding medium, *r* is the particle radius, *E* is the RMS electric field and *Re*[*K(ω*)] is the real part of the CM factor. The frequency-dependent CM factor in turn is given by:


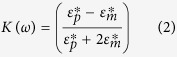


where 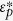 is the complex permittivity of the particle (i.e. a cell or a polystyrene bead) and 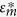 is the complex permittivity of the medium. All CM-factor calculations assume an effective medium permittivity of 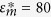.

When the permittivity (or polarizability) of the particle is greater than that of the medium, 0 < *K(ω*) ≤ 1, the particle will experience positive dielectrophoresis. Conversely, when the polarizability of the medium is greater than the particle, 0 > *K(ω*) ≥ −0.5, the particle will experience negative dielectrophoresis.

### Yeast two-shell model

In this model, the complex permittivity of the cell, 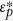, is given by:


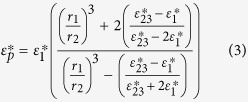


and


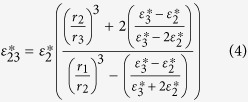


where radius *r*_1_ is the total average cell radius; *r*_2_ = *r*_1_ − *d*_1_ (where *d*_1_ is the cell wall thickness); and *r*_3_ = *r*_2_ − *d*_2_ (where *d*_2_ is the cell membrane thickness). 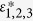 are the complex permittivities of the cell wall, membrane and cell interior, respectively, and are calculated using:


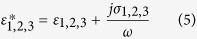


where *σ*_1,2,3_ and *ε*_1,2,3_ are the respective conductivity and real permittivity values (in multiples of *ε*_0_ = 8.854×10^−12 ^Fm^−1^), and *ω* is the angular frequency, which is related to the SAW frequency, *f*, by *ω* = 2*πf*.

The values for viable and non-viable yeast-cell CM calculations are given in Table 1.

### Mammalian single-shell model

In this model, the complex permittivity of the cell, 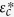, is given by:


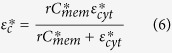


where 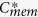 is the complex specific membrane capacitance, *r* is the cell radius and 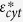 is the complex permittivity of the cytoplasm. 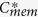 is given by:


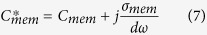


in which *C*_*mem*_ is the real part of the specific membrane capacitance, *σ*_*mem*_ is the membrane conductivity, *d* is the membrane thickness and *ω* is the angular frequency, which is related to the SAW frequency, *f*, by *ω* = 2*πf*. Finally, 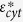 is given by:


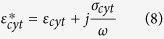


where *ε*_*cyt*_ is the real permittivity of the cytoplasm, and *σ*_*cyt*_ is the cytoplasm conductivity. The values for viable and non-viable yeast-cell CM calculations are given in Table 2.

Calculations were performed assuming an effective medium permittivity of 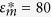.

### Latex bead solid sphere model

At high (>1 MHz) frequencies, the conductivity of latex beads, *σ*_*p*_, is dominated by their surface charge and is given by^44^:


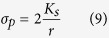


where *r* is the particle radius, and *K*_*s*_ is the particle surface conductivity:


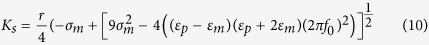


where *f*_0_ = 1 MHz is the crossover frequency for the latex spheres. The complex permittivity of the latex spheres is therefore given by:


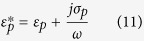


The values used for calculations are given in Table 3.

Latex beads therefore experience negative DEP for all values of media conductivity (*ε*_*m*_) at the high (>1 MHz) frequencies used for SAW-DEP separation (Supplementary Fig. 4).

### Finite element modelling

To confirm the absence of significant Joule heating and mechanical coupling effects within the microfluidic channel, three dimensional finite element simulations were performed using COMSOL Multiphysics 5.2a (COMSOL Inc., Burlington, USA). To reduce computational time, the device symmetry was exploited by imposing a symmetry plane equidistant between opposing IDTs, normal to the direction of the SAW propagation (Supplementary Fig. S2).

The geometry width was set at 5 mm (symmetric about the SAW propagation axis), beyond the extent of which the mechanical and electrical energies are negligible. The transducers were modelled as massless boundaries on the piezoelectric material. The mechanical wave propagation (described by the displacement field, **U**_Sub_) and associated electric field (described by the electrical potential field, V_Sub_) inside the substrate were modelled by combining the frequency domain forms of the wave equation (eq. 12), charge conservation equation (eq. 13), and the linear piezoelectric constitutive relations (eq. 14).


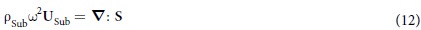



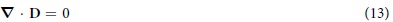



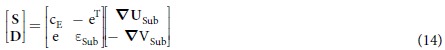


S, D, c_E_, e and *ε*_Sub_ respectively represent the mechanical stress tensor in the material, the electric displacement field, the elasticity matrix, the piezoelectric coupling matrix and the substrate permittivity. The transfer of mechanical and electrical energy from the piezoelectric substrate into both the underlying circuit and the PDMS fluidic channel, were simulated within using eqs 1 and 2, and the uncoupled field variables (**S** = c_E_**∇U**_Sub_; **D** = −ε_Sub_**∇**V_Sub_). The transfer of electrical energy from the substrate into the conducting liquid inside the microfluidic channel (described by the electrical potential field V_Ch_) was simulated using the frequency domain form of the current conservation equation (eq. 15).


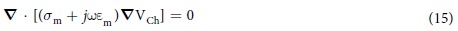


where *σ*_m_ and *ε*_m_ are the electrical conductivity and permittivity of the fluid, respectively. The temperature, T, induced by Joule heating, was solved over the entire device geometry using the steady state energy equation (eq. 16).


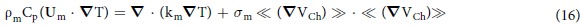


where ρ_m_, C_p_ and k_m_ are, respectively, the density, specific heat capacity and thermal conductivity of the fluid medium. The velocity field **U**_m_ was solved using the incompressible linear Navier-Stokes equations for a Newtonian fluid in a low Reynolds number microfluidic channel (eqs 17 and 18).


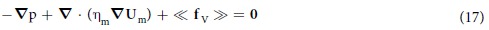



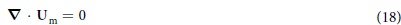


where η_m_ is the dynamic viscosity of the fluid, and 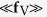
contains the time averaged volume forces induced inside the liquid by the acoustic pressure field induced within the fluid from the mechanical SAW component. This energy transfer results into a first order perturbation of the bulk pressure field, flow field and temperature field inside the fluid, denoted by the primed quantities in the frequency domain forms of the thermo-viscous acoustic equations (eqs 19,20,21,22).


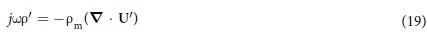



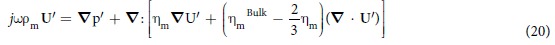



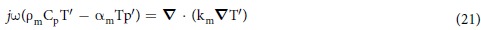



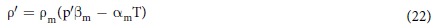


Here, η_m_^Bulk^ represents the bulk viscosity of the fluid medium, while α_m_ and β_m_ respectively represent the coefficient of thermal expansion and isothermal compressibility of the fluid medium. The volume force obtained from the perturbation fields is then expressed as follows:


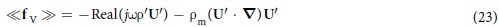


This approach of solving the effective fluid flow field within the channel as a superposition of the applied pressure driven flow and the secondary perturbations induced by acoustic radiation is directly adapted from the theoretical work by Muller *et al*.^45^.

The time dependent motion of a polarizable particle of radius “r” suspended inside the microfluidic channel is governed by a balance of the acoustic radiation force, the DEP force, sedimentation force and the Stokes viscous drag resistance. It can be expressed as follows:


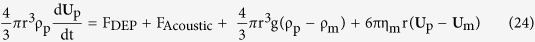


where ρ_p_ and **U**_p_ respectively denote the particle density and particle velocity vector. The last 2 terms on the RHS of eq. 13 respectively represent the sedimentation force (where g is the gravitational acceleration of −9.81 m/s^2^) and the Stokes viscous drag resistance on the particle in the fluid medium (where **U**_m_ is the fluid flow velocity obtained from eq. 6). F_DEP_ and F_Acoustic_ are the DEP and acoustic radiation forces acting on the particle, respectively. F_Acoustic_ can be expressed as^46^:


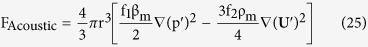


where the primed quantities are the acoustically induced flow perturbations as described previously. The scattering functions f_1_ and f_2_ can be expressed as:


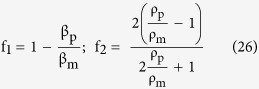


where β_p_ is the particle isothermal compressibility.

### Boundary Conditions

Low-reflection boundaries were set at the device perimeter to ensure generation of standing waves within the device arose solely from the interaction of the waves generated by the individual IDTs. The electric field equations were closed by specifying the applied sinusoidal power across each of the transducers, and by setting the device boundaries to be electrically open. Since these equations were solved collectively over the piezoelectric substrate, PDMS and accompanying circuit board, a continuity of field variables at the interfaces was inherently imposed in the model. Likewise, the coupling of mechanical displacements within the fluid medium and the piezoelectric substrate was also imposed inherently within the system. The continuity conditions for electrical potential and electric displacement across the fluid-piezoelectric interface however, were imposed as manual constraints, and served as the closure conditions for current-conservation within the fluid. Finally, fluid flow closed by imposing a flow rate at the inlet and atmospheric pressure at the outlet, whilst the temperature equations were closed by assuming thermal outflow conditions along the side walls of the geometry and a natural convection condition (h = 10 W/m^2 ^K) over the remaining boundaries.

### Simulation and Material Properties

The model was solved using the “Piezoelectric Devices”, “Thermoacoustic-Structure Interaction”, “Creeping Flow” and “Heat Transfer” modules in COMSOL. The electrical conductivity, permittivity dynamic viscosity and bulk viscosity of the medium were modelled as functions of temperature as below^47^,^48^:


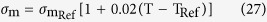



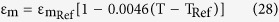



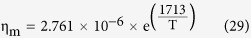



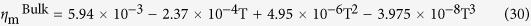


Here *σ*_m Ref_ and *ε*_m Ref_ respectively represent the electrical conductivity and permittivity of the fluid at a reference temperature of T_Ref_ (293 K or 20 °C in this model). The orientation of the piezoelectric crystal cut relative to the Cartesian coordinate system was incorporated in the model by defining an Euler coordinate system with the Z-X-Z rotation conventions for the substrate. This ensured that the above matrices were inherently transformed while solving the equations to generate the shear SAWs required. For particle simulations, all particles were initially at rest. A structured mesh was employed in which the mesh size was set to 1/40^th^ of the channel width within the microfluidic channel (to sufficiently resolve the fluid flow), and to 1/5^th^ of the SAW wavelength outside the fluidic channel (to resolve the SAW waveform). The final grid contained ~0.2 million finite elements, translating to ~2.5 million degrees of freedom for computation. Simulations were performed using the Amazon EC2 (Elastic Cloud Computing) service. COMSOL library values for all material properties, alongside anisotropic material property matrices, were used for all simulations.

## Additional Information

**Accession codes:** The data associated with this paper are openly available from the University of Leeds data repository: http://doi.org/10.5518/75.

**How to cite this article:** Smith, A. J. *et al*. Rapid cell separation with minimal manipulation for autologous cell therapies. *Sci. Rep.* **7**, 41872; doi: 10.1038/srep41872 (2017).

**Publisher's note:** Springer Nature remains neutral with regard to jurisdictional claims in published maps and institutional affiliations.

## Supplementary Material

Supplementary Information

## Figures and Tables

**Table 1:** Clausius-Mossotti parameters for yeast cells.

Parameter	Viable cells	Non-viable cells
*ε*_1_	60	60
*ε*_2_	6	6
*ε*_3_	50	78
*σ*_1_	0.014 S/m	0.0015 S/m
*σ*_2_	2.5 × 10^−7^ S/m	1.6 × 10^−4^ S/m
*σ*_3_	0.520 S/m	0.007 S/m
*r*_1_	4 μm	2.5 μm
*d*_1_	0.22 μm	0.22 μm
*d*_2_	8 nm	8 nm

Values used to calculate the CM-factor for viable and non-viable yeast cells as a function of SAW-DEP frequency and medium conductivity^23^.

**Table 2:** Clausius-Mossotti parameters for human cells.

Parameter	Viable cells	Non-viable cells
*σ*_*mem*_	3 × 10^−6^ S/m	3 × 10^−6^ S/m
*C*_*mem*_	9 mF/m^2^	9 mF/m^2^
*ε*_*cyt*_	57	57
*σ*_*cyt*_	0.52 S/m	0.0052 S/m
*r*	7.5 μm	7.5 μm
*d*	8 nm	8 nm

Values used to calculate the CM-factor for viable and non-viable human erythrocytes as a function of SAW-DEP frequency and medium conductivity^40^.

**Table 3:** Clausius-Mossotti parameters for latex spheres.

Parameter	Latex beads
*r*	0.5 μm
*ε*_*m*_	80
*ε*_*p*_	2.56
*r*	7.5 μm
*d*	8 nm

Values used to calculate the CM-factor for latex spheres as a function of SAW-DEP frequency and medium conductivity^44^.
