# Supplementary material for: Rapid cell separation with minimal manipulation for autologous cell therapies
Source: Sci Rep. 2017 Feb 2;7:41872. doi: 10.1038/srep41872 (PMC5288801; doi:10.1038/srep41872)
Supplement: Supplementary Information [file srep41872-s1.pdf]

# Rapid cell separation with minimal manipulation for autologous cell therapies

Alban J. Smith[1], Richard D. O'Rorke[1,3], Akshay Kale[1], Roberts Rimsa[1], Matthew J. Tomlinson[2], Jennifer Kirkham[2], A. Giles Davies[1], Christoph Wälti[1], Christopher D. Wood[1*]

[1]School of Electronic and Electrical Engineering, University of Leeds, Woodhouse Lane, Leeds, West Yorkshire, LS2 9JT

[2]University of Leeds, Department of Oral Biology, Leeds School of Dentistry, Leeds, UK.

[3](Current address): Division of Materials Technology, Nanyang Technological University, 50 Nanyang Avenue, Singapore 639798.

*C.D.Wood@leeds.ac.uk

**Supplementary information**

**Supplementary Figure S1**

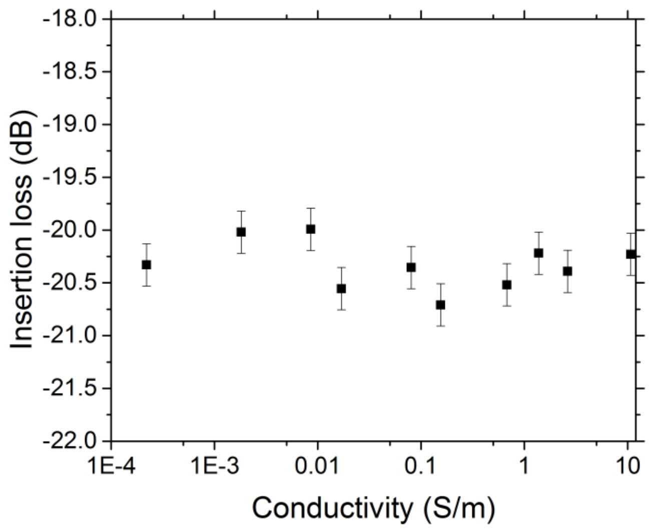


**Fig. S1: SAW-DEP insertion loss as a function of medium conductivity**: The insertion loss for a 10 MHz device was measured as a function of medium conductivity using a network analyser. No appreciable change was observed, suggesting that no increase in coupling between the SAW and the medium occurs as the conductivity is raised.

**Supplementary Figure S2**

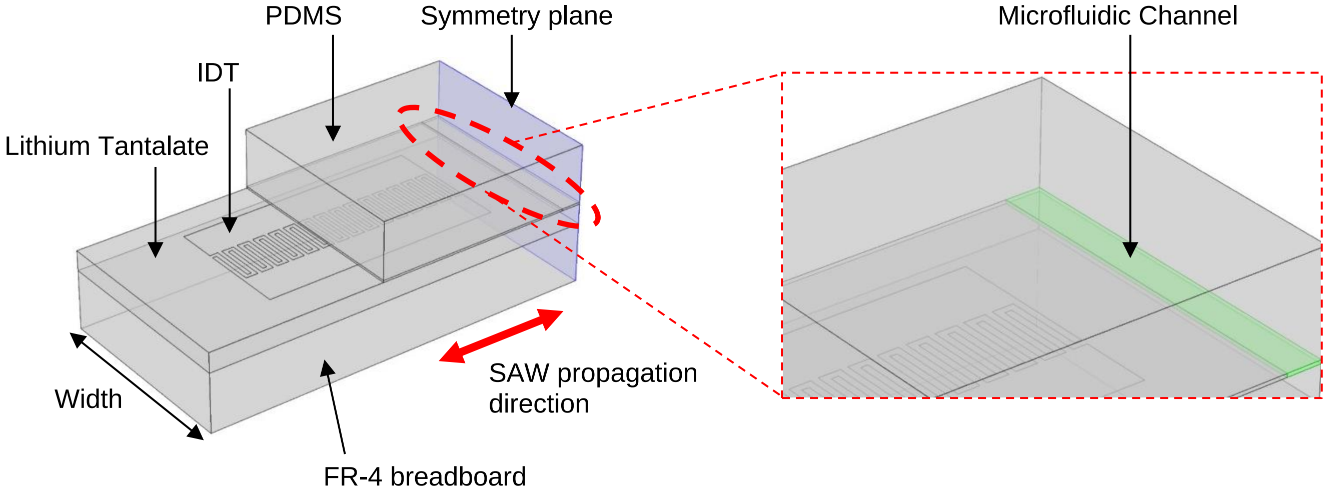


**Fig. S2: 3D finite element model of SAW-DEP device:** Finite element simulations were conducted using the full 3D model shown here. Device symmetry was exploited to reduce simulation time by imposing a symmetry boundary. The device width was set as 2.5 mm either side of the IDT centre (along the SAW-propagation axis), beyond which no mechanical or electrical oscillations were observed. Inset is a magnified view of the microfluidic channel formed in PDMS.

**Supplementary Figure S3**

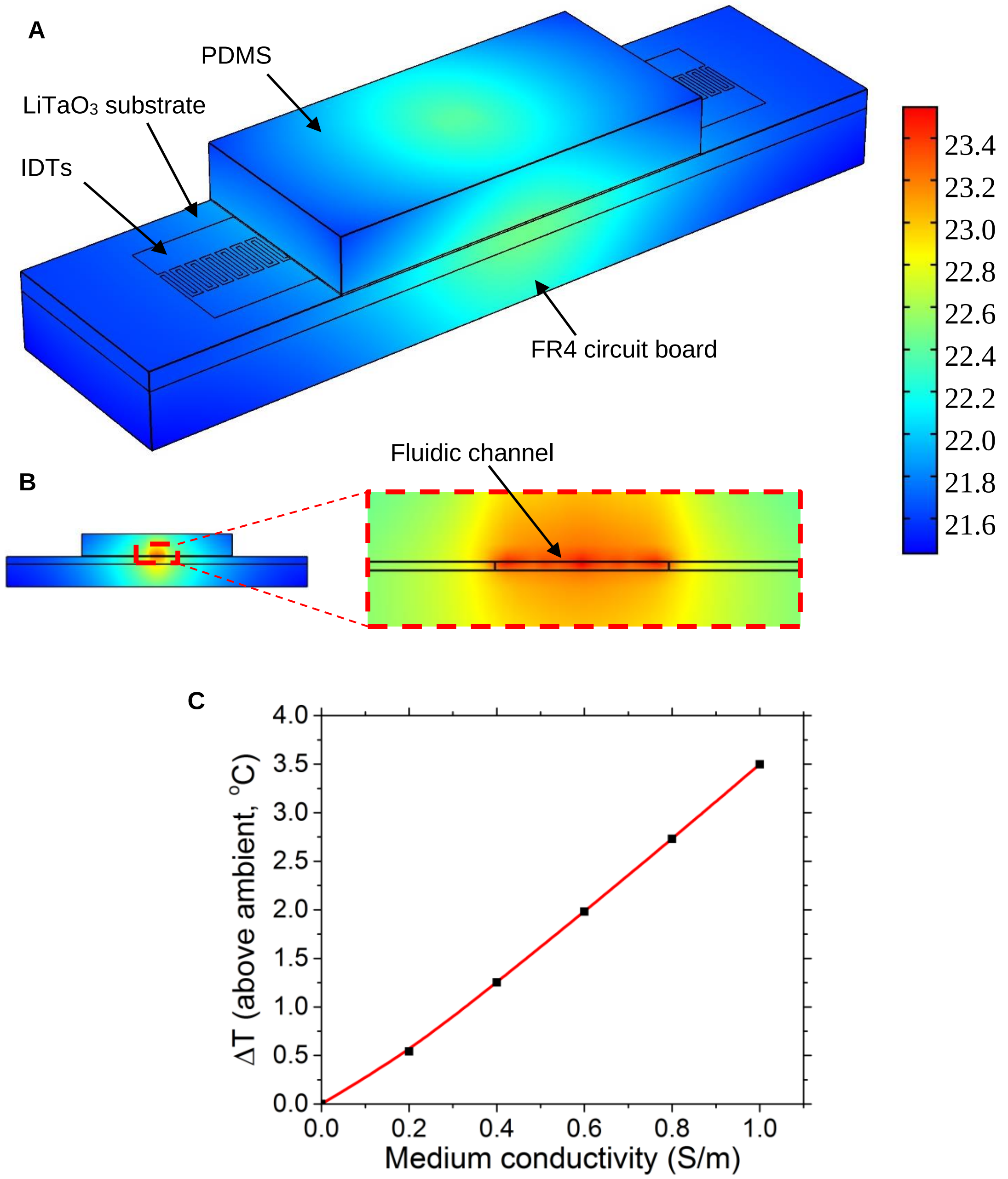


**Fig. S3: Simulations of device temperature as a function of medium conductivity**: The steady state, absolute device temperature (in degrees Celsius) was calculated using a full 3D finite element simulation of the device (in COMSOL) for (**A**) the full device, including fluidic channel, piezoelectric substrate and the supporting FR4 circuit board; (**B**) a cross section of the device, with an inset showing a magnified cross section of the fluidic channel. (**C**) shows the maximum temperature increase above ambient conditions for fluid conductivities varying from 0 – 1 S/m

**Supplementary Figure S4**

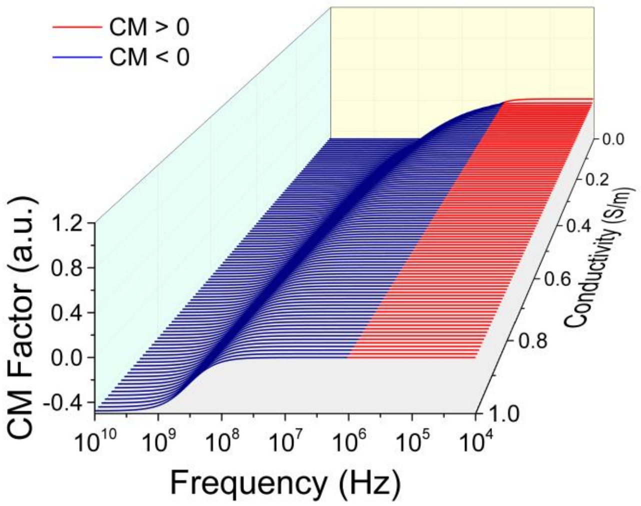


**Fig. S4: Clausius-Mossotti factor for latex spheres:** Calculations of the frequency dependent CM-factor for 1-µm-diameter latex spheres for medium conductivities between 0 – 1 S/m. At frequencies > 1 MHz, the CM factor is always negative and the spheres therefore undergo nDEP.

**Supplementary Figure S5**

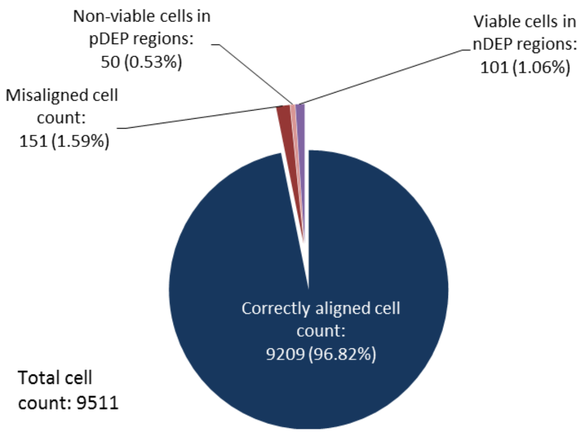


**Fig. S5: Yeast cell alignment statistics:** A sample population of ~ 10,000 yeast cells, in which non-viable cells were stained dark blue using Trypan blue, were imaged during SAW-DEP separation and subsequently the number and location of cells were counted. Percentages are calculated using the total number of yeast cells counted. Cell misalignment owing to adhesion to the channel walls / other cells was < 3%, whilst enrichment of viable cells within the pDEP regions was > 99%**.**

**Supplementary Figure S6**

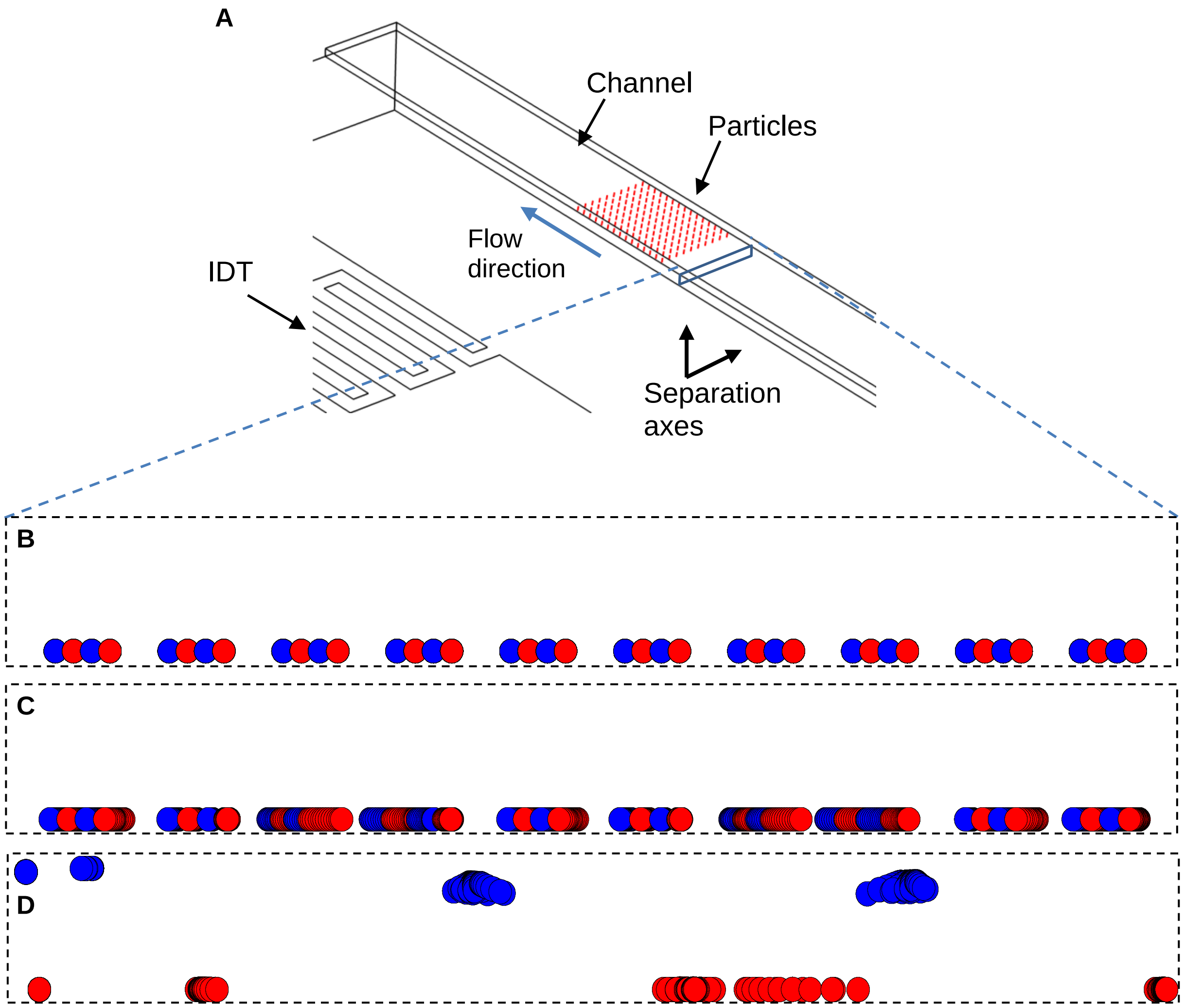


**Fig. S6: DEP and acoustic-radiation alignment forces:** In order to assess the contribution, separately, of dielectrophoretic and acoustophoretic forces generated by the SH-SAW standing wave on particle separation, two, full-3D finite element simulations were performed in which the mechanical and electrical coupling were isolated and assessed individually. An array of 10 µm solid particles, 50% of which had a CM factor of 0.5 (red), and 50% -0.5 (blue), were suspended in fluid of conductivity of 0.2 S/m, and distributed across the channel in (**A**) as a 3D image, and in (**B**) a projected view looking along the channel in the direction of flow (corresponding to time t = 0 for both simulations). The distribution of particles across the channel at t = 7 seconds (after activation of the SH-SAW) is shown for (**C**) acoustophoretic and (**D**) dielectrophoretic coupling, separately. Although a small, horizontal particle displacement caused by acoustophoretic forces is observed, no separation of pDEP from nDEP particles occurs in any direction under application only of acoustophoretic forces. Conversely, significant, CM-factor dependent particle separation occurs under application of DEP-only forces, both laterally and vertically within the channel (corresponding to nDEP particles being pushed away from the surface and aligning at areas with lowest electric field gradient). The results confirm that any acoustophoretic contribution to cell separation can therefore be deemed negligible, and separate is truly DEP-driven.

**Supplementary Figure S7**

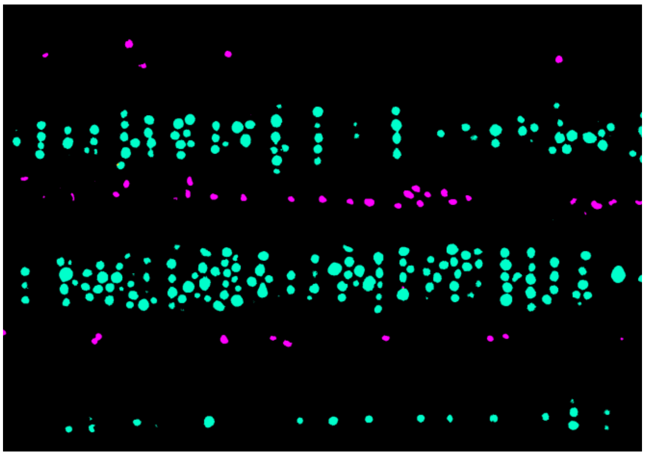

**Fig. S7: Binary separation of viable from non-viable DPSCs:** A fluorescence image of Calcein-AM stained viable DPSCs (cyan) overlaid with PI-stained non-viable DPSCs (purple) in a media conductivity of 0.05 S/m. The viable and non-viable DPSCs are separated with 100% efficiency into pDEP and nDEP bands, respectively, using SAW-DEP operating at 10 MHz.
